# YOLOv7-DWS: tea bud recognition and detection network in multi-density environment via improved YOLOv7

**DOI:** 10.3389/fpls.2024.1503033

**Published:** 2025-01-07

**Authors:** Xiaoming Wang, Zhenlong Wu, Guannan Xiao, Chongyang Han, Cheng Fang

**Affiliations:** ^1^ Chengdu Polytechnic, Innovation and Practice Base for Postdoctors, Chengdu, Sichuan, China; ^2^ Sichuan Provincial Engineering Research Center of Thermoelectric Materials and Devices, Chengdu, Sichuan, China; ^3^ College of Engineering, South China Agricultural University, Guangzhou, China

**Keywords:** tea buds, images recognition, multi-density, object detection, YOLOv7, deep learning

## Abstract

**Introduction:**

Accurate detection and recognition of tea bud images can drive advances in intelligent harvesting machinery for tea gardens and technology for tea bud pests and diseases. In order to realize the recognition and grading of tea buds in a complex multi-density tea garden environment.

**Methods:**

This paper proposes an improved YOLOv7 object detection algorithm, called YOLOv7-DWS, which focuses on improving the accuracy of tea recognition. First, we make a series of improvements to the YOLOv7 algorithm, including decouple head to replace the head of YOLOv7, to enhance the feature extraction ability of the model and optimize the class decision logic. The problem of simultaneous detection and classification of one-bud-one-leaf and one-bud-two-leaves of tea was solved. Secondly, a new loss function WiseIoU is proposed for the loss function in YOLOv7, which improves the accuracy of the model. Finally, we evaluate different attention mechanisms to enhance the model’s focus on key features.

**Results and discussion:**

The experimental results show that the improved YOLOv7 algorithm has significantly improved over the original algorithm in all evaluation indexes, especially in the $R_{\text{Tea}}$ (+6.2%) and mAP@0.5 (+7.7%). From the results, the algorithm in this paper helps to provide a new perspective and possibility for the field of tea image recognition.

## 1 Introduction

As one of the most popular drinks in the world, the production and quality control process of tea requires high-precision inspection technology (Bai et al., 2022). Accurate tea image recognition and detection is of great value for tea production, quality assessment, pest prevention and other fields (Xue et al., 2023; Zhao et al., 2022). Early tea inspection mainly relied on manual inspection, but this method was inefficient and accuracy was affected by manual skill and fatigue (Ngugi et al., 2021). With the development of computer vision and deep learning technology, tea detection technology has also changed significantly (Liu and Wang, 2021).

The traditional tea detection methods mainly include manual inspection and mechanical screening technology (Hu et al., 2021; Tian et al., 2022). Manual inspection usually relies on experienced workers observing the tea leaves through the eyes to identify the type and quality of the tea (Lin et al., 2022). Mechanical screening is to separate different sizes of tea by the size of the sieve (Pruteanu et al., 2023). In addition, some image processing techniques, including edge detection, threshold segmentation and color analysis, are also widely used in tea recognition projects. These techniques can realize automatic recognition of tea images to a certain extent (Lu et al., 2023; Li et al., 2020). Karunasena et al. developed a machine learning method for tea bud recognition, they used the histogram gradient (HOG) method for tea buds with an overall recognition accuracy of 55% for tea buds between 0 mm and 40 mm in length (Karunasena and Priyankara, 2020). Bojie et al. introduced a tea bud point recognition process based on RGB images, using the HSI color transform and HSV spatial transform and segmenting the tea bud pictures, and the image of tea buds can be obtained by setting the threshold to merge the three channel components, which has a good effect in practical application (Bojie et al., 2019). However, in order to meet the requirements of fast and accurate recognition in the vision system of picking robots, deep learning techniques bring possibilities.

In recent years, the development of deep learning technology has brought new possibilities to tea detection. Deep learning can automatically learn features in images, avoiding the complexity of manual feature extraction and improving the accuracy of tea detection (Li et al., 2021; Xiong et al., 2020; Yang et al., 2021). Yang et al. proposed an improved Yolo-v3 algorithm for tea tree new shoot picking points. The method used image pyramid structure to integrate tea trees of different levels, and the K-means method was used to cluster the size of the target frame. Finally, a high-quality tea tree selection point image dataset was constructed. The model accuracy rate reached 90%, and the prediction of tea tree selection point was roughly realized (Yang et al., 2019). Further, the inference speed of the model is an index that must be considered in object detection algorithms. In order to solve the problem of slow inference speed of existing detection models. Zhang et al. proposed a light tea tree crown growth detection model (TS-YOLO) based on YOLOv4, with a size of 11.78 M and an improved detection speed of 11.68 FPS. This model is easier to deploy quickly (Zhang et al., 2023). In order to achieve the detection of small targets of tea buds, Wang et al. used the attention mechanism to improve the YOLOv5 tea bud recognition network. More detailed tea bud information was obtained, and the false detection and omission caused by different tea bud sizes were reduced. Experimental results showed that the accuracy rate (P) of the proposed method was 93.38%, and it could accurately detect the tea bud area (Wang et al., 2024). It can be seen that the object detection model based on deep learning technology has been applied to the problem of tea bud recognition. Some scholars have studied the object detection model of tea bud, focusing on improving the reasoning speed and lightweight of the model, but the problem of tea bud classification under multi-density has been ignored. In the real tea garden environment, because of the small size and high density of tea buds, the accuracy of target detection model is very difficult. The purpose of this paper is to realize the stratified detection of tea buds in multi-density tea garden environment, including the detection of a single leaf of a bud and two leaves of a bud at the same time, and to develop a new tea bud target detection model.

It is noteworthy that the grading of tea buds includes one-bud-one-leaf and one-bud-two-leaves, and how to achieve the simultaneous detection and recognition of one bud and one bud and two leaves in tea bud images will strongly promote the development process of tea bud picking robots. Base on the powerful tool of deep learning, this work proposed an improved YOLOv7 detection algorithm to solve the problem of one-bud-one-leaf and one-bud-two-leaves detection and classification. The main contributions of this paper can be summarized as follows:

1. A multi-density tea dataset was constructed, which included two types: one-bud-one-leaf and one-bud-two-leaves.
2. The enhanced tea leaf detection algorithm, YOLOv7-DWS was proposed, which has achieved varying degrees of improvement in detection performance across different densities, with the mAP@0.5:0.95 metric experiencing respective boosts of 5.5%, 6.5%, and 8.2% for low, medium, and high densities.
3. The experimental results demonstrated that the proposed method was highly effective in detecting tea leaves under various density conditions. Through ablation studies, it was revealed that, compared to the original YOLOv7 algorithm, the improved YOLOv7-DWS significantly enhances tea leaf detection with a 6.2% increase in $R_{Tea}$ and a 7.7% rise in mAP@0.5.

The rest of this article is arranged as follows. The second part provides the basic principles of the relevant models and algorithms used for training, the relevant information of the data set and the relevant evaluation criteria. The experimental results are analyzed and visualized in the third part. The fourth part discusses the algorithm of this question and some directions of future optimization research. The conclusion of this paper is presented in the fifth part.

## 2 Materials and methods

### 2.1 Data collection

**Figure 1:**
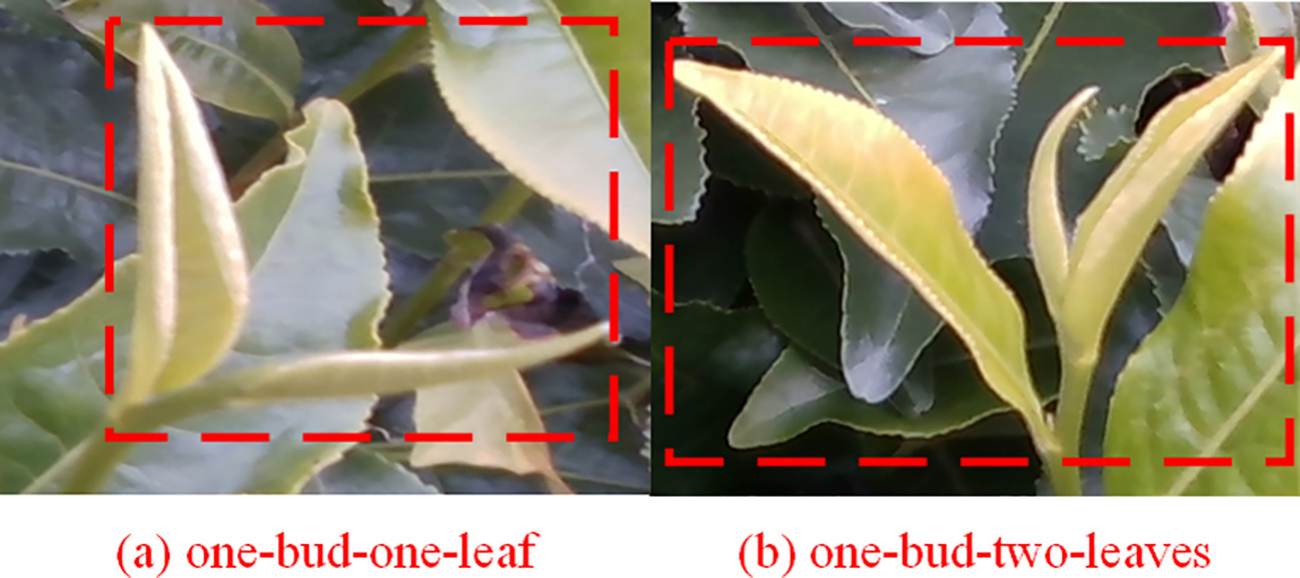
Diagram of different types of picking Yinghong No. 9 tea leaves. **(A)** one-bud-one-leaf; **(B)** one-bud-two-leaves.

The data pertaining to tea was amassed between March 2 and April 28, 2023. It was gathered from several tea gardens located in Yingde City, Guangdong Province, PR China. The specific variety of tea leaves is Yinghong No. 9, a tea variety extensively cultivated in this location. Yinghong No. 9, known for its distinctive characteristics and high-quality taste, enjoys a good reputation in both domestic and international tea markets. We chose this variety for our research with the intention to deeply analyze and detect its growth under various conditions. This will potentially improve the efficiency and accuracy of intelligent harvesting equipment. The authors specifically chose to work with tea leaves of the type “one-bud-one-leaf” and “one-bud-two-leaves”. As shown in **Figure 1**, These types are particularly significant in tea harvesting as they often represent the ideal harvesting stage for many tea varieties, offering the best balance between quality and quantity. A high-resolution image capturing sensor (Realsense435 camera) was employed to gather detailed visuals of these tea leaf types. This camera, capable of a resolution of 1920*1080, allowed us to capture extremely detailed images that significantly benefited our analysis. All captured images were saved in the.jpg format and transferred to a computer via a USB connection, thus preserving their high-quality state while ensuring convenient accessibility for the research team. The data collected by this sensor served as the basis for the further stages of our study, including image processing and feature extraction.

### 2.2 Dataset

**Figure 2:**
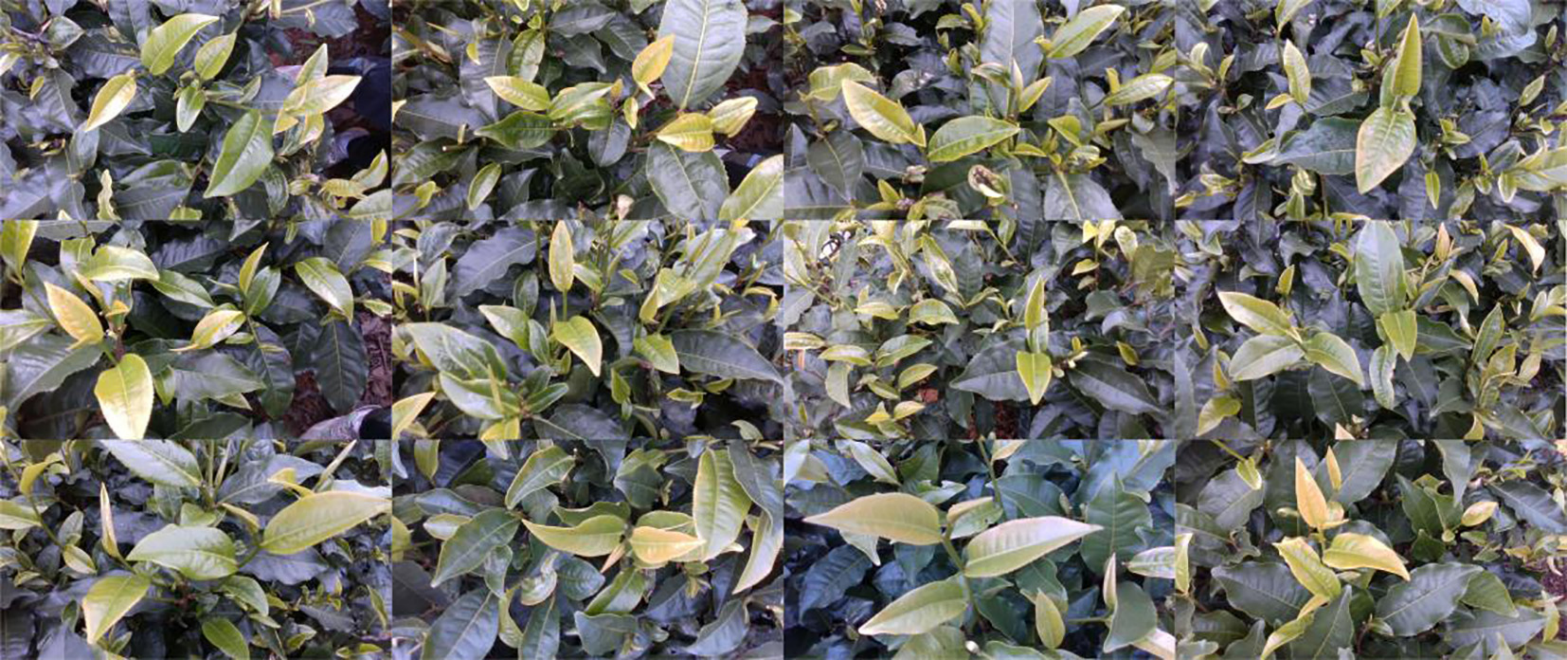
Multi-density images of a partial tea dataset.

In order to increase the richness of the image and improve the generalization ability of the model, we captured the tea bud image from multiple distances under different backgrounds and lighting conditions (morning and afternoon), taking into account different angles (30 degrees, 60 degrees and 90 degrees). Finally, 945 original images were collected, and part of the dataset images are shown in **Figure 2**. In addition, in order to reduce the factor interference of tea bud image background and improve the feature extraction ability of the model, data enhancement methods such as increasing noise, darkening/brightening image, stretching and rotating image were adopted. The hue is randomly adjusted by 1.5%, the saturation by 80% and the value by 45% of the image. Moreover, the image is set to flip up and down with a 50% probability, and the degree of random is set to 30%. Finally, the image data is annotated with Labelme and stored in PASCAL VOC format. Among them, the “one bud, one leaf” type label is designated as “tea11”, while “tea12” is used to indicate the “one bud, two leaves” type.

### 2.3 Network structure for detection and classification of tea buds

**Figure 3:**
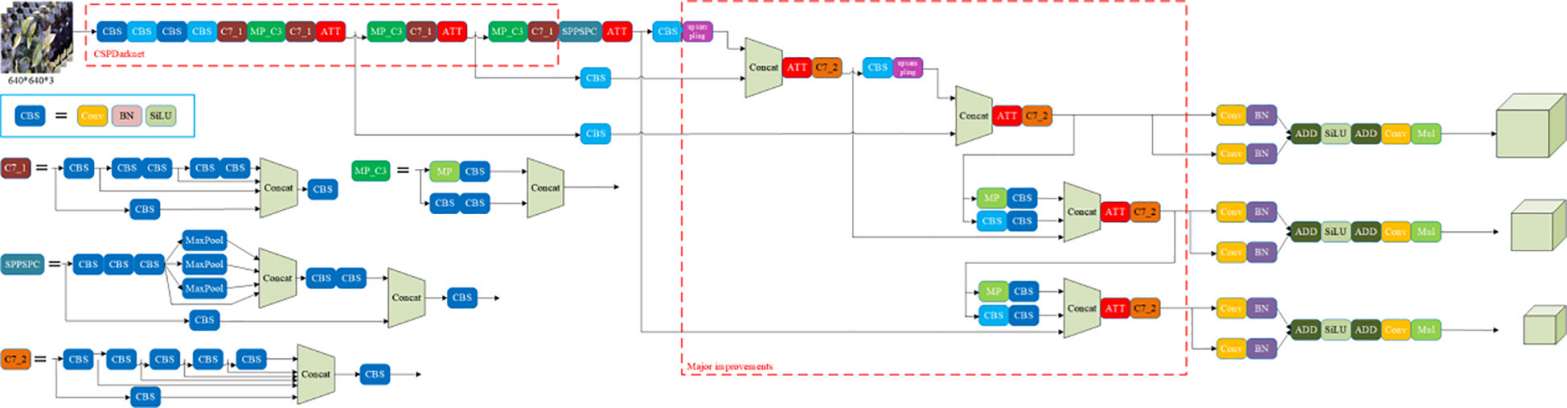
Network structure of YOLOv7-DWS model.

YOLOv7 was introduced by Alexey Bochkovskiy in 2022 (Zhou et al., 2022; Ali et al., 2022). It is mainly composed of a backbone network, neck network, head network, and loss function. First, YOLOv7 employs a backbone network called CSPDarknet for image feature extraction. CSPDarknet serves as the backbone of the YOLOv7 model, enhancing feature extraction by employing Cross Stage Partial (CSP) connections. This technique aims to reduce the computational load while maintaining accuracy, making it more efficient for real-time applications. Specifically, CSPDarknet helps in splitting feature maps and re-merging them to improve gradient flow and reduce memory usage, thus optimizing the network for object detection tasks (Zhang et al., 2022). Next, YOLOv7 utilizes an SPP-PAN neck network that compresses and integrates the output features from the backbone network, thereby optimizing them for object detection tasks. This network incorporates pyramid pooling and progressive aggregation strategies to bolster detection performance. Finally, YOLOv7’s head network, YOLO-FPN, is used for predicting object locations and classes. This network features adaptive feature pyramids and a feature aggregation module, improving detection accuracy and speed. **Figure 3** shows the network structure diagram of YOLOv7-DWS improved by YOLOv7.

The original YOLOv7 utilizes a loss function called GIoU-L1 to measure the distance between predicted boxes and ground truth boxes. This loss function is an improved version of IoU loss, and it takes into account factors such as the position, size, and shape of the predicted boxes, aiming for enhanced detection performance. As shown in **Figure 3**, our main improvements include the replacement of coupled heads in Major improvement areas, the replacement of IoULoss, and the addition of SimAM.

### 2.4 Improvements in network structure

#### 2.4.1 Decoupled head

Decoupled head is a concept used within the context of object detection using deep learning. It refers to an architectural modification in the head of a neural network to decouple the various prediction tasks, allowing the network to focus on different aspects of the prediction separately (Liu et al., 2023).

In traditional object detection architectures, the prediction head of the neural network is responsible for predicting several attributes of the object, such as its class, bounding box coordinates, and possibly additional attributes like object pose or segmentation mask. These predictions are often entangled within the same network layers, meaning that the same set of neurons is responsible for handling multiple prediction tasks.

**Figure 4:**
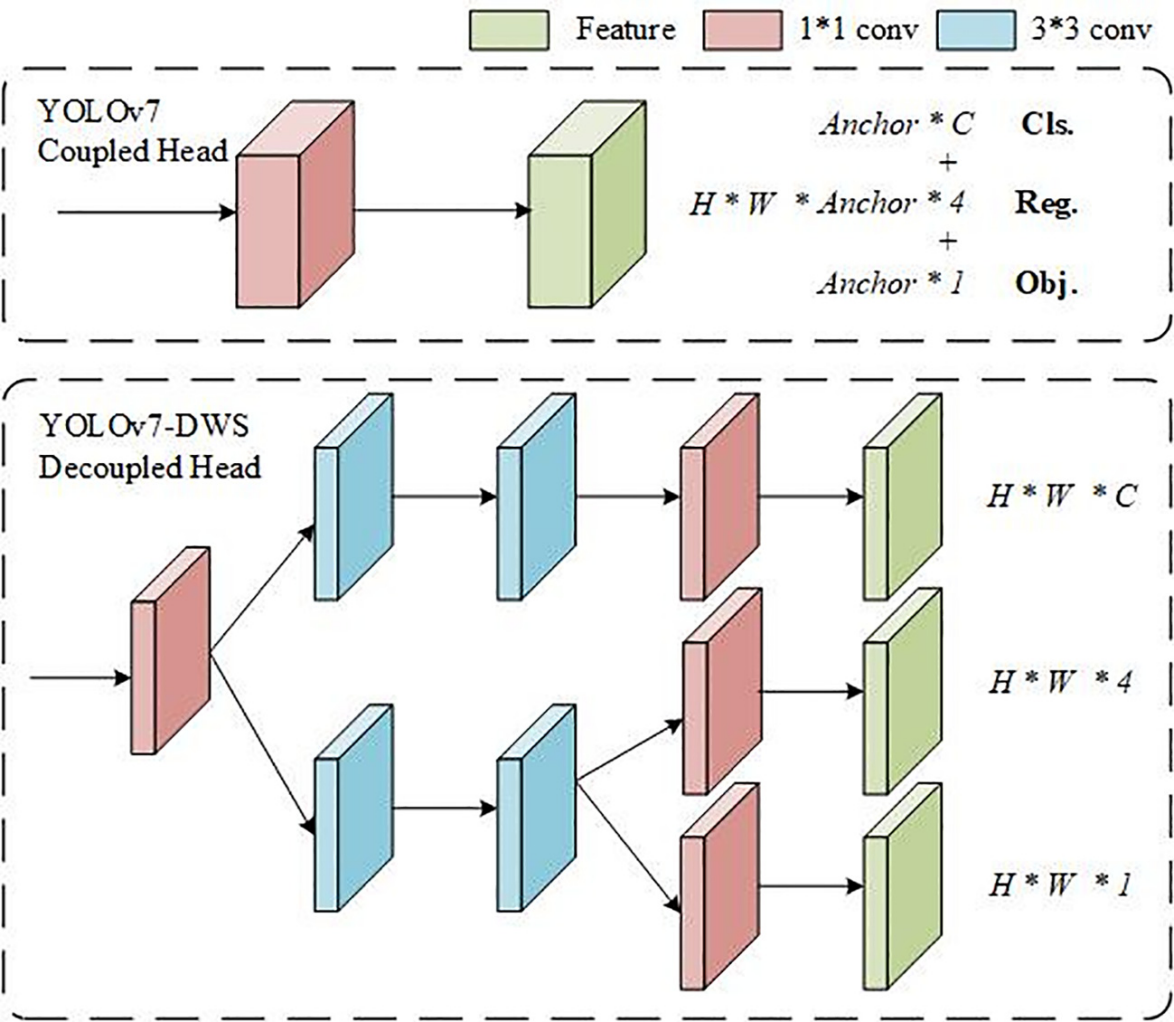
Decoupled head structure schematic.

As shown in **Figure 4**, Decoupled head approach aims to overcome this limitation by decoupling or separating the prediction tasks into distinct sets of neurons or layers within the prediction head. This allows the network to learn features and representations that are specifically tailored to each prediction task, such as classification or bounding box regression, without interference from other tasks.

#### 2.4.2 WiseIoU

Training data inevitably contains low-quality examples. As such, geometric measurements like distance and aspect ratio tend to intensify the penalties on these poor-quality samples, which in turn can degrade the generalization performance of the model (Li et al., 2023). An effective loss function should mitigate the penalties from geometric measurements when the anchor boxes sufficiently overlap with the target boxes. Minimizing excessive interference during training can enhance the model’s generalization capabilities. Building on this principle, a distance attention mechanism is developed through distance measurement, leading to the creation of WiseIoU, as depicted in (Equations 1, 2):


(1) $${ℒ}_{WIoU}={ℛ}_{WIoU}{ℒ}_{IoU}$$

(2) $${ℛ}_{WIoU}=exp\left(\frac{{\left(x-x_{gt}\right)}^{2}+{\left(y-y_{gt}\right)}^{2}}{{\left(W_{g}^{2}+H_{g}^{2}\right)}^{*}}\right)$$

Where $W_{g}$ and $H_{g}$ represent the width and height of the anchor box, and $W_{g}^{2}+H_{g}^{2}$ represent the diagonal length of the box. The superscript * indicates a detachment operation, which is used to prevent ${ℛ}_{WIoU}$ from producing gradients that hinder convergence.

#### 2.4.3 SimAM

**Figure 5:**
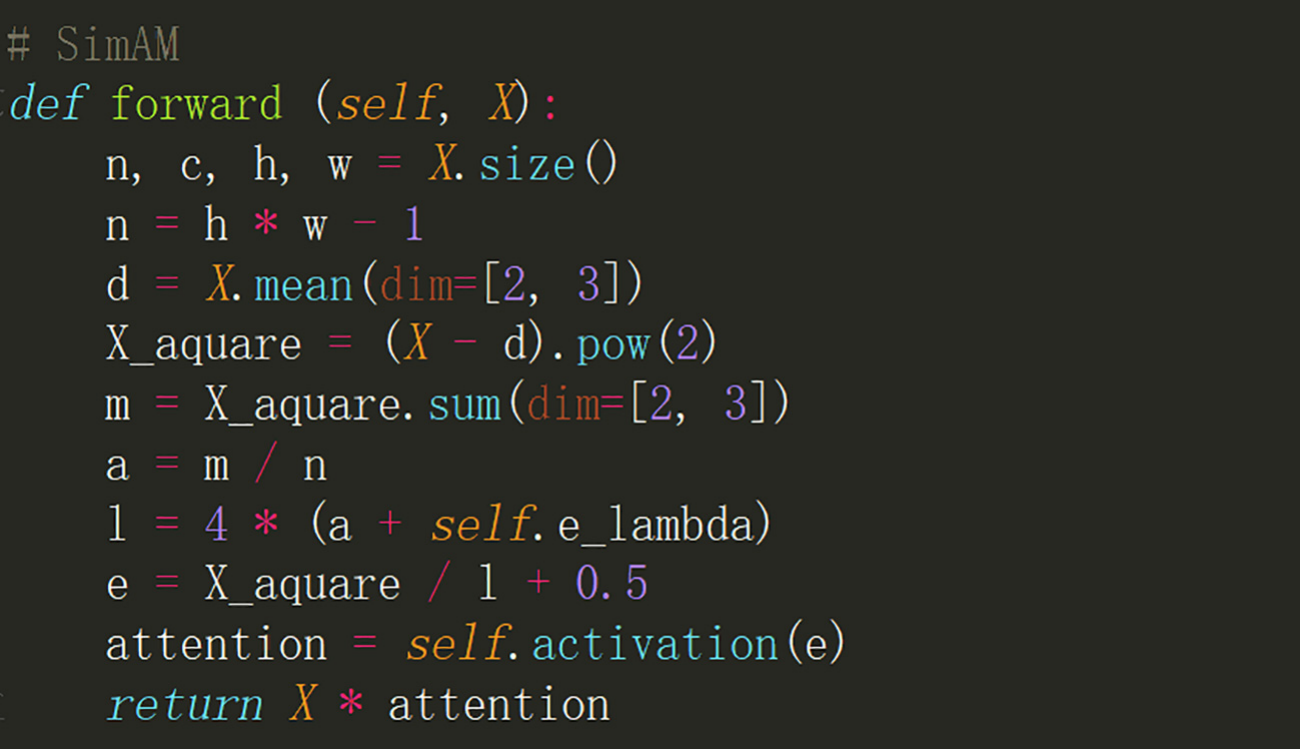
A PyTorch way of implementing SimAM.

SimAM takes inspiration from the concept of energy exploration in neuroscience, which distinguishes the importance of neurons, to access the attention mechanism within feature maps (You et al., 2023). The PyTorch implementation of SimAM is illustrated in **Figure 5**.

The integration of SimAM brings about a dynamic perspective into the neural network by selectively focusing on crucial aspects within the feature maps. This selective attention enables the model to learn highly expressive representations which are vital for complex tasks such as object detection, segmentation, and classification. As SimAM is grounded in the principles of neuroscience, it aligns well with the cognitive processes, allowing for more intuitive and human-like interpretation of data.

### 2.5 Density analysis

The number of tea leaves in different images is different, which results in different tea density in the images. In order to see the performance of the algorithm under different densities, we propose a tea density distribution index (TDDI) analysis method to analyze the detection performance of tea images with different densities. Since there are two types of one-bud-one-leaf and one-bud-two-leaves in the image, we need to calculate the area of the real rectangular box of the two types of tea respectively, as shown in (Equations 3, 4):


(3) $$Area_{N}={\left(H_{N}\;*\;W_{N}\right)}_{tea11N}$$

(4) $$Area_{M}={\left(H_{M}\;*\;W_{M}\right)}_{tea11M}$$

Where $Area_{N}$ and $Area_{M}$ represent the area of the Nth one-bud-one-leaf and the *M*th one-bud-two-leaves, respectively. $H_{N}$ and $W_{N}$ represent the height and width of the *N*th one-bud-one-leaf rectangular box, $H_{M}$ and $W_{M}$ represent the height and width of the *M*th one-bud-two-leaves rectangular box.

Then calculate the total area of each type of tea, as shown in (Equations 5, 6):


(5) $$Total_{tea11}=\sum_{i=1}^{N}Area_{N}$$

(6) $$Total_{tea12}=\sum_{\text{j}=1}^{\text{M}}Area_{M}$$

Where $Total_{tea11}$ and $Total_{tea12}$ represent the total area of the one-bud-one-leaf and the one-bud-two-leaves.

Considering that there will be overlapping occlusion between tea leaves, we then calculate the union of the areas of the real rectangular boxes of the two types of tea leaves, as shown in (Equations 7, 8):


(7) $$Union_{tea11}=Area_{1}\cup^{\;}Area_{2}\cup^{\;}\ldots \cup^{\;}Area_{N}$$

(8) $$Union_{tea12}=Area_{1}\cup^{\;}Area_{2}\cup^{\;}\ldots \cup^{\;}Area_{M}$$

Where $\;Union_{tea11}$ and $\;Union_{tea12}\;$ represent the union of the total area of the one-bud-one-leaf and the one-bud-two-leaves.

Then calculate the dense distribution of each tea type, as shown in (Equations 9, 10):


(9) $$TDDI_{tea11}=Union_{tea11}/Total_{tea11}$$

(10) $$TDDI_{tea12}=Union_{tea12}/Total_{tea12}$$

Where $\;TDDI_{tea11}\;$ and $TDDI_{tea12\;}$ represent the dense distribution index of the one-bud-one-leaf and the one-bud-two-leaves.

Finally, the overall density distribution index of the image is judged by combining these two density distribution indexes, as shown in Equation 11.


(11) $$\begin{array}{c} TDDI=TDDI_{tea11}\;*\;\left(Total_{tea11}/Area_{image}\right)\\ +TDDI_{tea12}\;*\;\left(Total_{tea12}/Area_{image}\right) \end{array}$$

Where TDDI represent the dense distribution index of tea in the whole image and $Area_{image}$ represent the total area of pixels in the image.

The formula takes into account the size difference between the different types of one-bud-one-leaf and one-bud-two-leaves, as well as the overlap between different tea leaves and the proportion of tea in the image, which can better reflect the dense distribution of tea in the image.

### 2.6 Evaluation metrics

In order to evaluate the performance of the proposed algorithm, several indicators were proposed in this study, including $P_{Tea}$ , $R_{Tea}$ , mAP@0.5, and mAP@0.5:0.95. $P_{Tea}$ is the proportion of truly correctly predicted samples (true positives) among all samples predicted as positive. $R_{Tea}$ is a metric used to evaluate the ability of a model to identify all relevant instances. These two indicators can be obtained from (Equations 12, 13):


(12) $$P_{Tea}=TP/\left(TP+FP\right)$$

(13) $$R_{Tea}=TP/\left(TP+FN\right)$$

where TP is true positive; FP is false positive; FN is a false negative.

Mean average precision ( mAP ) serves as a measure of the overall performance of a model’s detection results across all categories, as shown in Equation 14:


(14) $$mAP=\;\sum^{\;}AP_{i}/C$$

Where $AP_{i}$ represent the ith category of *AP*, C is the total number of categories.

For mAP@0.5, when calculating *AP*, we only consider predictions with an intersection over union (IoU) greater than 0.5. For mAP@0.5:0.95, when calculating *AP*, we consider predictions with IoU values at 0.5, 0.55,…, 0.95, respectively. Then average all the calculated *APs*, and indicators can be obtained from Equation 15:


(15) $$mAP@0.5:0.95=\;\sum^{\;}\left(AP_{i}@r\right)/\left(C\cdot R\right)$$

Where $AP_{i}@r$ was the AP for the ith class under the prediction with an IoU of *r*, and *R* is the number of IoU thresholds. The mAP@0.5:0.95 takes into account the degree of match between the predicted bounding boxes and the actual bounding boxes under different thresholds. By selecting different thresholds, it considers the performance of the model under various matching criteria.

## 3 Results and analysis

### 3.1 Experimental setting

In this study, the software and hardware environment used in the experiment was shown in **Table 1**.

**Table 1:** Hardware and software environments.

Hardware	CPU	I7-3960X@3.30GHz
	RAM	16G
	GPU	NVIDIA GTX 1080TI
Software	Operating System	Windows 10 Pro
	CUDA	11.3.1
	CUDNN	8.4.0
	Python	3.8
	PyTorch	1.12.1

**Figure 6:**
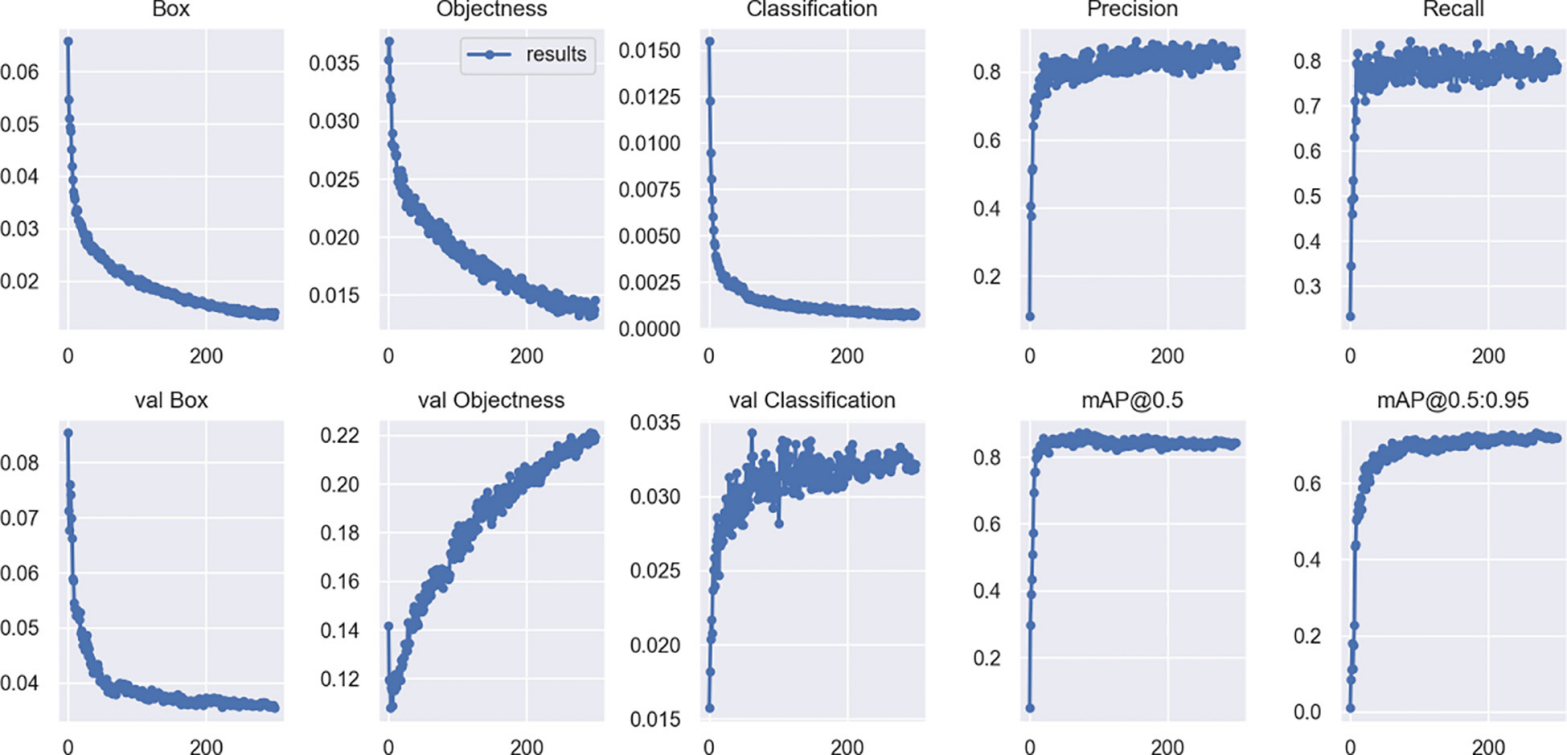
Visual validation of different attention mechanisms.

As shown in **Table 1**, the computer used for training and testing in this study was configured with: i7-3960X@3.30GHz CPU, 16G RAM, single NVIDIA GTX 1080TI GPU, and software environment: Windows 10 operating system, Python 3.8, PyTorch 1.12.1, CUDA 11.3.1, CUDNN 8.4.0. The size off the image used for algorithm input was 640*640 pixels. The ratio of the training set to the test set was 9:1. A total of 300 rounds were trained, using Stochastic Gradient Descent (SGD) as the optimizer. SGD enables faster parameter updates as it uses only a single training example per iteration, and its noisy updates can sometimes help the model escape local minima (Wu et al., 2024; Fang et al., 2024; Guo et al., 2021). **Figure 6** shows the changes of various indicators of the YOLOv7 model in the training process.

The training and validation loss of the model decreased rapidly in the first 80 times, and slowly decreased in the following 80 to 250 times, and basically stabilized at about 250 times.

### 3.2 Comparative of IoU experimental

In this section, we will discuss the effect of the improvement of different algorithm strategies on the detection of one-bud-one-leaf and one-bud-two-leaves, and conduct ablation experiments on decoupled head, different loss functions, and attention mechanisms. Indicators used for evaluation included *P_Tea_*, *R_Tea_*, mAP@0.5, mAP@0.5:0.95, parameters and detection speed.

In this paper, we selected YOLOv7 as the base network and considered replacing the original IoU with SIoU, DIoU, GIoU, EIoU, and WiseIoU for comparative experiments, thereby obtaining the IoU with the best recognition accuracy.

As shown in **Table 2**, YOLOv7+WiseIoU has the overall balanced detection performance, specifically speaking, The model employing the WiseIoU loss function achieved a precision ( $P_{Tea}$ ) of 86.3%, which is considerably higher by 5.9%, 1.5%, 6.0%, and 3.6% compared to the models using SIoU, DIoU, GIoU, and EIoU loss functions respectively.

**Table 2:** Comparative results of IoU experiments (%).

Algorithm	Class	*P_Tea_*	*R_Tea_*	mAP@0.5	mAP@0.5:0.95
YOLOv7+SIoU	All Tea11 Tea12	80.2 79.6 80.7	80.6 79.5 81.6	83.5 80.7 86.2	71.5 67.0 76.0
YOLOv7+DIoU	All Tea11 Tea12	84.8 84.2 85.3	77.0 75.0 79.0	83.0 81.9 84.2	71.0 67.9 74.1
YOLOv7+GIoU	All Tea11 Tea12	80.3 78.7 81.9	79.1 76.6 81.6	81.5 79.0 84.0	69.5 66.1 72.9
YOLOv7+EIoU	All Tea11 Tea12	82.7 82.0 83.4	78.4 77.4 79.4	83.1 81.8 84.3	71.5 68.7 74.3
YOLOv7+WiseIoU	All Tea11 Tea12	86.3 85.7 86.8	74.9 73.5 76.4	83.1 81.2 85.0	72.0 68.2 75.8

This significant improvement in precision with the use of the WiseIoU loss function suggests that it has a more effective capability in handling the complexities involved in tea leaf detection. The higher precision reflects the model’s ability to correctly identify and classify tea leaves, which is critical for practical applications such as automated harvesting and quality assessment.

Considering these advantages, adopting WiseIoU as the loss function can be instrumental in enhancing the robustness and reliability of tea leaf detection systems. WiseIoU, by providing a gradient gain allocation strategy, focuses on anchors of ordinary quality, thereby making the overall performance of the detector more balanced.

### 3.3 Comparative of attention mechanism experimental

In this section, we aim to enhance the algorithm’s efficacy in tea leaf recognition by incorporating various attention mechanisms. We contemplate augmenting the original network by separately integrating SEATT, SimAM, BiFormer, TripleATT, CoTATT, and ShuffleAttention. Through comparative experiments, we seek to identify the attention mechanism that yields the highest recognition accuracy.

After integrating each of these attention mechanisms, rigorous validation procedures were conducted. The experiments were designed to measure not only the $P_{Tea}$ , $R_{Tea}$ , mAP@0.5, mAP@0.5:0.95 but also other relevant metrics such as parameters and speed, providing a more holistic view of each mechanism’s performance.

As shown in **Table 3**, SimAM stands out as the most effective in boosting the overall performance of the model in tea leaf detection. The inclusion of SimAM in the model leads to a 4.2% increase in $P_{Tea}$ for tea11 detection, a 4.6% improvement in $R_{Tea}$ , a 7.9% rise in mAP@0.5, and a 6.7% elevation in mAP@0.5:0.95. Notably, these improvements are achieved with almost no change in the number of parameters and the detection speed. The experimental results highlight that employing the SimAM attention mechanism can effectively enhance tea leaf detection.

**Table 3:** Comparative results of attention mechanism experiments (%).

Algorithm	Class	*P_Tea_*	*R_Tea_*	mAP@0.5	mAP@0.5:0.95	Parameters	Speed(ms)
YOLOv7	Tea11 Tea12	81.3 88.1	75.2 80.1	76.9 85.9	66.1 76.5	36,487,166	14.5
YOLOv7+SEATT	Tea11 Tea12	81.7 83.3	79.3 82.3	84.8 88.0	70.8 79.3	36,890,110	15.5
YOLOv7+SimAM	Tea11 Tea12	85.5 87.7	79.8 82.0	84.8 87.4	72.8 78.7	36,488,702	15.4
YOLOv7+Biformer	Tea11 Tea12	81.2 84.1	77.8 83.1	83.9 89.1	69.3 78.9	49,394,686	26.7
YOLOv7+TripleATT	Tea11 Tea12	79.8 84.7	78.7 82.8	81.5 87.7	69.4 79.2	36,490,802	17.4
YOLOv7+CoTATT	Tea11 Tea12	82.6 82.2	79.3 83.5	86.0 88.7	71.7 78.6	64,648,190	23.4
YOLOv7+ ShuffleAttention	Tea11 Tea12	83.2 84.7	78.7 83.2	84.2 88.4	71.6 79.3	62,646,526	16.7

### 3.4 Ablation experimental

To evaluate the efficacy of the proposed algorithm in tea leaf detection, we conducted ablation studies on decoupled Head, the WiseIoU loss function, and the SimAM attention mechanism. As depicted in **Table 4**, the experimental results indicated that our approach, which incorporates structural and strategic enhancements, is effective.

**Table 4:** Results of ablation experiments (%).

Number	+ Decoupled	+ WiseIoU	+ SimAM	R_Tea_	mAP@0.5	Parameters	Speed(ms)
0				77.7	81.4	36,487,166	14.5
1	√			78.7	83.7	62,644,990	18.8
2		√		74.9	83.1	36,487,166	14.9
3			√	80.9	86.1	36,488,702	15.4
4	√	√	√	83.9	89.1	62,646,526	20.2

Compared to the original YOLOv7, the improved YOLOv7-DWS boasts a significant enhancement, with a 6.2% increase in *R_Tea_* and a 7.7% rise in mAP@0.5. These significant improvements underscore the importance of employing an integrative approach to optimization for enhancing the tea leaf detection algorithm. The refinements in YOLOv7-DWS have not only elevated the model’s accuracy but also laid the foundation for its application in more complex and diverse scenarios. It’s noteworthy that by integrating the WiseIoU loss function and SimAM attention mechanism, our model exhibits higher robustness in processing tea leaf images under various conditions. This is crucial for practical applications such as automated harvesting and tea leaf quality assessment.

### 3.5 Comparison of results under different density conditions

In order to further verify the actual effect of our improved algorithm under different tea densities, we divided the test pictures into low density, medium density and high density. The experimental results was shown in **Table 5**.

**Table 5:** Comparison of detection results under different densities (%).

Density	Algorithm	Class	*P_Tea_*	*R_Tea_*	mAP@0.5	mAP@0.5:0.95
Low	YOLOv7	All Tea11 Tea12	76.9 76.2 77.7	79.6 76.2 82.9	81.2 77.8 84.5	72.7 67.0 78.4
	YOLOv7-DWS	All Tea11 Tea12	81.4 80.7 82.1	86.5 84.4 88.6	86.7 84.5 88.8	73.0 69.2 76.7
Medium	YOLOv7	All Tea11 Tea12	88.6 86.5 90.7	79.7 78.4 81.0	85.1 82.9 87.4	72.4 68.5 76.2
	YOLOv7-DWS	All Tea11 Tea12	88.5 88.8 88.1	83.1 82.2 84.0	91.6 91.1 92.0	76.6 73.3 79.9
High	YOLOv7	All Tea11 Tea12	74.4 71.4 77.3	81.7 83.3 80.0	79.1 69.3 89.0	64.9 53.8 75.9
	YOLOv7-DWS	All Tea11 Tea12	90.4 80.9 99.8	75.5 71.0 80.0	86.0 76.2 95.8	73.1 62.6 83.5

As shown in **Table 5**, the improved YOLOv7-DWS has achieved commendable performance in detection across various densities. For low density, *P_Tea_* improved from 76.9% to 81.4%, an increase of 4.5%; *R_Tea_* rose from 79.6% to 86.5%, a gain of 6.9%; mAP@0.5 increased from 81.2% to 86.7%, a rise of 5.5%; and mAP@0.5:0.95 inched up from 72.7% to 73.0%, a marginal improvement of 0.3%. In medium density, *R_Tea_* went up from 79.7% to 83.1%, an increase of 3.4%; mAP@0.5 escalated from 85.1% to 91.6%, a rise of 6.5%; and mAP@0.5:0.95 increased from 72.4% to 76.6%, a gain of 4.2%. For high density, *P_Tea_* rocketed from 74.4% to 90.4%, a significant surge of 16.0%; mAP@0.5 improved from 79.1% to 86.0%, a boost of 6.9%; and mAP@0.5:0.95 jumped from 64.9% to 73.1%, an improvement of 8.2%.

These significant improvements underscore that, through optimization and refinement, YOLOv7-DWS is adept at effectively enhancing the precision of tea leaf detection across various environmental densities. This augmented performance is vital for practical applications, as the growing conditions of tea leaves can substantially vary at different times and locations.

### 3.6 Model visualization analysis

**Figure 7:**
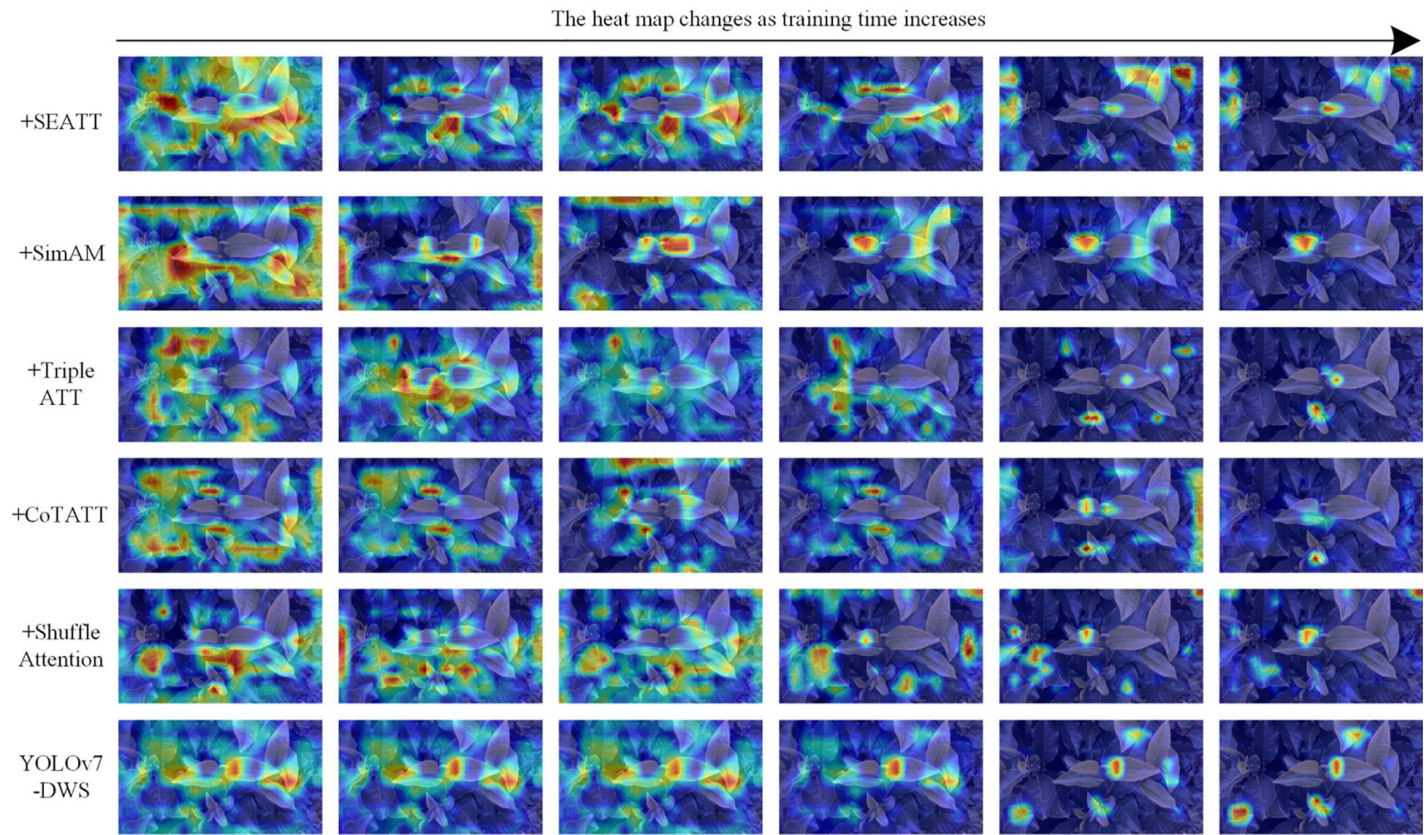
Visual validation of different attention mechanisms.

Deep neural networks, while adept at handling object detection tasks, often fall short in offering insights into which areas of the input they are focusing on. To address this limitation, this study employs heat map to contrast the visualization efficacy of the enhanced network. Heat map serve as a powerful tool to illustrate the areas of attention within the network, revealing the regions that the model deems significant for making its predictions (Yu et al., 2022). This not only adds a layer of transparency to the workings of deep neural networks but also aids in understanding and optimizing their performance. In this study, by integrating heat map, we can observe and compare how the original and the improved network focus on different areas of the tea leaf images. This comparison enables us to discern the advantages of the enhancements we integrated into the network and their impact on the attention mechanism. **Figure 7** shows the heat map changes of tea with the addition of different attention modules.

As depicted in **Figure 7**, as the training time progresses, the areas of focus in the heat map become increasingly concentrated. This concentration signifies that the model is gradually honing in on the relevant features and is likely developing a more refined understanding of the patterns within the data. As the model continues to evolve, this could potentially lead to better performance and improved prediction accuracy, particularly in complex tasks where discerning subtle features is crucial. The heat map serves as a valuable tool in visually tracking and comprehending the model’s learning trajectory.

**Figure 8:**
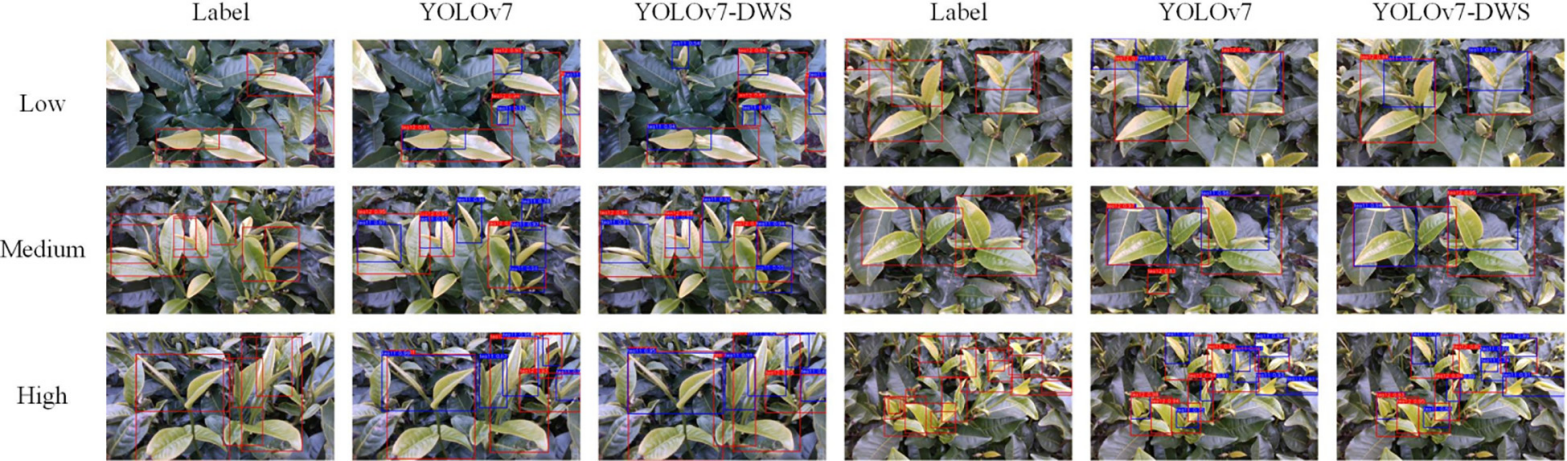
Visual verification of tea detection at different densities.

As shown in **Figure 8**, the improved YOLOv7-DWS achieves results that are closer to human annotations across different densities. This level of accuracy, akin to human annotations, not only reduces the need for human intervention but also provides a more reliable foundation for automated tools. By minimizing errors and improving accuracy, YOLOv7-DWS brings potential value to the tea industry, including optimizing harvest times, improving the quality of tea, and enhancing decision-making through precise data analysis.

Moreover, considering the seasonality and environmental changes in tea cultivation, having a detection algorithm capable of adapting to these variations is critically important. YOLOv7-DWS meets this requirement by delivering consistent high performance under various density conditions.

## 4 Discussion

This article introduces an algorithm called YOLOv7-DWS for tea leaf detection and classification. By refining and optimizing YOLOv7, YOLOv7-DWS manages to achieve results that are remarkably closer to human-annotated outcomes across various densities. This underscores the algorithm’s efficacy in emulating the keen insights of human experts.

Moving forward, the noteworthy achievements of YOLOv7-DWS can serve as a stepping stone for further advancements in agricultural technology. Its ability to closely mimic human expertise opens the doors for automation and precision in tea cultivation and quality control. Moreover, integrating YOLOv7-DWS into smart agricultural systems could revolutionize the way tea plantations operate, ultimately leading to enhanced productivity and sustainability.

Upon identifying the optimal approach for tea leaf detection and classification, the authors intend to use this as a foundation and draw inspiration from the concept of knowledge distillation in deep learning to embark on research focused on making the model lightweight (Laña et al., 2021). This will contribute to further advancing the deployment of the algorithm on edge devices. Specifically, the authors plan to construct a teacher-student framework. The high-accuracy model that has been trained will serve as the teacher model, guiding a lighter, student model through the learning process. The objective is to transfer the knowledge from the more complex, resource-intensive teacher model to the more streamlined student model without a significant loss in performance.

This distillation process will involve training the student model to mimic the behavior and outputs of the teacher model. Through this process, it is expected that the student model will acquire the ability to make similarly accurate predictions but with reduced computational requirements. Implementing such a lightweight model is particularly advantageous for deployment on edge devices, which are often constrained by limited resources. By reducing the model’s complexity without substantially compromising accuracy, it becomes feasible to integrate the algorithm into real-time applications on edge devices, thus providing a practical and efficient solution for tea leaf detection and classification in the field.

However, despite the promising aspects of YOLOv7-DWS and the planned knowledge distillation approach, there are still some challenges and areas that require further improvement. First and foremost, the diversity and size of the dataset used for training the model play a critical role in its performance. The current dataset may not encompass all the variations in tea leaf characteristics found globally. Thus, expanding the dataset to include a more diverse set of tea leaves, capturing different species, growth conditions, and geographical locations, would greatly enhance the model’s ability to generalize and maintain high accuracy in different scenarios (Zhang et al., 2023; Wu et al., 2022).

Additionally, it is essential to account for the robustness of the model in varying environmental conditions. For instance, the algorithm should be tested and tuned for performance under different lighting conditions, weather patterns, and levels of occlusion. This would make the model more adaptable and practical for real-world implementations where these variables can significantly affect the detection results (Lehnert et al., 2020).

Another area worth exploring is the integration of YOLOv7-DWS with other sensors and data sources. For example, incorporating information from soil sensors, weather data, and multispectral imagery could allow for a more comprehensive analysis of the tea crop health and quality (Ouhami et al., 2021). This multi-modal approach could lead to more informed and precise decision-making for tea cultivation.

Moreover, while knowledge distillation is a powerful technique for model optimization, it’s essential to carefully evaluate the trade-offs between model complexity and performance. There is a risk of losing some fine-grained information during the distillation process, which might impact the model’s ability to detect subtle variations in tea leaves. Developing methods to retain this granular information while still achieving model compression would be valuable (Liu et al., 2022).

In conclusion, YOLOv7-DWS represents a significant advancement in tea leaf detection and classification. However, by expanding the dataset, ensuring environmental robustness, integrating with other data sources, and carefully managing the trade-offs of model compression, further improvements can be achieved, pushing the boundaries of what is possible in smart agriculture for tea cultivation.

## 5 Conclusions

With the development of deep learning technology, the image recognition technology of tea is also improving. The tea recognition method based on deep learning achieves better performance and provides higher accuracy than the traditional tea recognition method. Secondly, this paper proposes a new tea object detection algorithm based on YOLOv7 algorithm (YOLOV7-DWS). The algorithm analyzed tea images in different density environments, and by comparing the experimental results, the following conclusions were drawn:

1. The inclusion of specialized optimization techniques within YOLOv7-DWS has shown to be critical in boosting the performance metrics, proving that tailored modifications can significantly impact the outcomes.
2. The improved YOLOv7-DWS has achieved commendable performance in detection across various densities. These significant improvements underscore that, through optimization and refinement, YOLOv7-DWS is adept at effectively enhancing the precision of tea leaf detection across various environmental densities.
3. The experimental results demonstrate that the proposed method is highly effective in detecting tea leaves under various density conditions. Through ablation studies, it was revealed that, compared to the original YOLOv7 algorithm, the improved YOLOv7-DWS significantly enhances tea leaf detection with a 6.2% increase in $R_{\text{Tea}}$ and a 7.7% rise in mAP@0.5.

The results show that our method can effectively detect tea under different density conditions. However, there is still a lot of work that we can continue to explore, and future work will expand the size of the dataset and test and validate it in richer scenarios to further optimize the algorithm. At the same time, we expect that the application of deep learning in tea detection will be more extensive, providing stronger support for tea production and quality control.

## Data Availability

The datasets generated for this study are available on request. Requests to access these datasets should be directed to Chongyang Han, hanchongyang7771@gmail.com.
